# Pathology and Practice of Medicine

**Published:** 1860-11

**Authors:** Richard J. Dunglison

**Affiliations:** One of the Physicians to the Pennsylvania Institution for the Instruction of the Blind, etc.


### PATHOLOGY AND PRACTICE OF MEDICINE.

BY RICHARD J. DUNGLISON, M.D.,

ONE OF THE PHYSICIANS TO THE PENNSYLVANIA INSTITUTION FOR THE INSTRUCTION OF THE BLIND, ETC.

1.—*Connection between Chorea and Acute Rheumatism.* By G. W. CHILD, M.D., etc. (London Lancet, Sept. 15, 1860, p. 260.)

Dr. Child details several interesting cases as illustrative of the fact that rheumatism and chorea are "hereditarily convertible"—that is, that rheumatism in the parent may descend as chorea to the child. We may appropriately refer to the observations which he makes upon the five cases which he describes as embracing a condensed history of what has already been recorded upon this subject. Since the year 1850, when the existence of a connection between rheumatism and chorea was announced by M. Sée of Paris, and in England by

Dr. Senhouse,* much attention has been given to the subject. M. Botrel, in the following year, went so far as to declare that chorea is nothing but "rheumatism of the nervous centres."†

Botrel's view, however, was not generally accepted; one of the latest French writers (M. Moynier‡) declares the connection to be merely accidental, though he himself, inconsistently enough, tells us that, out of thirty cases which he examined, the connection in question could be traced in eighteen. Dr. West§ has never once met with it, nor (he says) has M. Rilliet at Geneva. It is well known that both diseases are often hereditary; and Dr. Begbie was, we believe, the first to point out the existence of a family connection between the two—to show, that is, that it frequently happens that where one member of a family is subject to chorea, another is rheumatic. M. Botrel's theory seems to point to the conclusion that one and the same blood-poison may manifest itself, according to circumstances, as either chorea or as rheumatism. Whatever may be thought of the theory, the cases described by Dr. Child are suggestive, and may lead to further useful inquiries.

II.—*Difficulties in the Diagnosis and Prognosis of Mental Diseases.* By C. M. BURNETT, M.D. (British Medical Journal, Aug. 4, 1860, p. 600.)

It seems appropriate at a time when the work of one of the most distinguished ornaments of the profession (Dr. Winslow) is attracting deserved attention, to refer briefly to remarks upon kindred topics to those embraced in it. Dr. Burnett has read before the Reading (Eng.) Pathological Society a paper upon difficulties which beset the path of the psychopathic inquirer. His object, according to his own language, is to take one or two of the more prominent signs of mental disturbance, and to follow those signs as they are to be observed in the various forms of mental disease. He examines first the subject of *delirium*, constituting not a disease, but merely a symptom, and only a symptom, whether we speak of it as acute or chronic delirium. Very different forms of cerebral disease may be characterized by this symptom, but the nature of the delirium is different in each, as in arachnitis and acute mania; yet two different forms of delirium may exist in the same disease.

Dr. Burnett then devotes attention to the *pulse*, and to its importance as an element in forming our prognosis. The pulse is always accelerated in acute mania at some period in the twenty-four hours more than at others. We may rest much of our prognosis confidently upon the pulse in such cases, if we bear in mind that the danger is indicated by the frequency in the return and duration of its acceleration. As long as there is a distinct subsiding in the frequency of the pulse, which generally occurs soon after the active paroxysm has passed off, we may hopefully anticipate a favorable issue. Rarely do we find, where there is real danger, the pulse, as in other cases, rising and falling with the paroxysm; but, on the contrary, we have a continuous and more or less accele-

* See Canstatt's Jahrsbericht, 1850, vol. iv.

† Ibid., 1851, vol. iv. ‡ Ibid., 1858, vol. iii. p. 44.

§ Diseases of Children, 4th edit., p. 206.

rated vascular action ; and this rule applies as well to the puerperal form of acute mania as to the acute mania arising from other causes. Dr. Burnett has unlimited confidence in the pulse as a means of prognosis in cerebral affections,—frequently regarded in other diseases as at times a fallacious guide. Several cases are detailed to warrant this confidence, in one of which he is anxious to show that in that kind of delirium which accompanies arachnitis, the variations in the frequency of the pulse are equally great as in the delirium of mania, and when those variations can be detected, the prognosis in the one, as the other, may be alike favorably formed.

In alluding to the occasional suddenness of invasion of cerebral disorders, the writer calls attention to the fact that, in spite of the intense functional disturbance, the changes of structure are by no means proportionate. So sudden is the attack, indeed, that we may well doubt sometimes whether the cerebral structures can have anything to do with it. Yet the explosion may be merely the manifestation of undetected causes which might escape the eye of the morbid-anatomist. The closer we watch our case, and compare the vascular and somatic disturbances by which they are accompanied, the more certainly shall we be assured that these degrees of delirium, whether in its complete or its incomplete form, and these marked changes in the character and frequency of the pulse, are closely and accurately dependent upon somatic changes going on at different parts of the organization. We must recollect that the state of the cerebral tissues and of the blood is not a fixed one, and therefore if the disease has been induced by a trifling exciting cause, it shows the structure of the brain to be less prepared for the assault. You see this well illustrated by observing what different effects the same quantity, or nearly the same, of some diffusible stimulant has upon different persons. It is the close study of these morbid phenomena that forms the groundwork of our most valuable and accurate diagnosis. Blood conveying to the brain not only a deficient amount of nutrient material, but some abnormal substitute for that material, must obviously fail to effect those healthy psychological phenomena, which can strictly belong only to tissues brought together in normal proportions. The more these proportions deviate from their healthy standard, the more unable is the brain to recognize truthfully the objects and phenomena of the outer world.

We are able to trace the rapid succession of exalted ideas, in the delirium of mania, to the presence of those substances in excess, which form, in their normal proportions, a large part of the chemical constituents of the brain. It is in the rapid succession of ideas we notice in some of the acute forms of mania, that we must all have been able, at times, to detect a gradual increase of incoherence, till all power of recognition, and even consciousness, seems to be lost. Those who suppose that because incoherence and unconsciousness are more complete in the acute cases than they are in some of the chronic and intermittent forms of the disease, they do not exist at all in these last, are probably not aware of the fact that a temporary abolition of the understanding, sufficient to destroy healthy mental gestation, takes place in both kinds of mania ; and they are still less prepared to recognize the presence of delirium, or its near relative, in many chronic diseases of the mind.

The delirium of drunkenness, Dr. Burnett remarks, is often mixed up with cases of chronic mania; and it is worth while to observe how far all those cases which present such a marked periodical character may not be cases of this kind, where the excitement produces first the incautious taking of the spirits, till at last it cannot be resisted. It may not be necessary to incarcerate the ordinary case of delirium tremens, and the patient may lapse again into a similar state, but in such cases as those first referred to, in which spirit has been taken as the effect of periodical or intermittent mania, these persons will, under judicious confinement, not only recover their minds, but their lives also will have probably been saved.

Such are some of the points of difficulty which are elucidated in the cases furnished by Dr. Burnett, and which receive ample consideration in the remarks made by him.

III.—*Pleurisy, Pericarditis, and Disease of Liver, supervening in advanced Caries of the Spine.* Communicated by EDWARD B. GRAY, M.B., Oxon. (Medical Times and Gazette, June 30, 1860, p. 7.)

A lad of eighteen years of age was admitted into the Radcliffe Infirmary at Oxford, with angular curvature of the lumbar spine, and a superficial abscess on the left hip, which burst about a week after admission, and discharged several ounces of thin pus. A few weeks afterwards he was seized with urgent dyspnœa, cough, and sharp pain in the left hypochondrium, and on the next day after the appearance of these symptoms, the skin of the whole body began to turn yellow. The urine was tinged with bile, and albuminous, but contained no blood-cells or kidney-casts. The severity of the chest symptoms gradually abated, auscultation exhibiting dullness and impaired respiration, with a crackling friction sound in the lower left lateral region of the chest.

The patient gradually sank, and post-mortem examination revealed the presence of a layer of recently effused, soft, villous lymph in the pericardium; old pleuritic adhesions on the right side; recent pleurisy, with abundant effusion of lymph and serum on the left side; the liver enlarged and in a state of extreme fatty degeneration, and the bodies of the four upper lumbar vertebræ completely destroyed. The bodies of the twelfth dorsal and fifth lumbar vertebræ were in apposition; their adjacent ends were carious, and, by straightening the spine, the slight gap between them could be considerably widened. The abscess went from the gap in the vertebræ, in the direction of the left psoas muscle, (very little of which remained,) down to the brim of the pelvis. There it took two courses: the one going through the abdominal wall into the left groin, and thence round on to the hip; the other descending into the pelvis in front of the denuded sacrum, and forming a cul-de-sac behind and somewhat to the left of the rectum.

In summing up the history of the case, the following pertinent remarks are made. There is an external abscess dependent on carious vertebræ. The abscess breaks and remains open. For the next month there is no irritative fever, nor a single bad symptom; then, in the course apparently of a few hours, and without obvious cause, we find severe inflammation set up in one pleura; this is very soon complicated with jaundice, and in eighteen days the patient dies. When the pericarditis came on is uncertain, but, from the post-mortem appearances, one would conclude it to be of more recent date than the pleurisy. What is the probable explanation of this series of morbid phenomena? In the absence of any exciting cause, either local or external, (as exposure to cold, etc.) to account for them, it seems reasonable to attribute them to pyæmia, or blood-poison of some kind.

The case is of interest as showing to what a state of things the spinal cord can accommodate itself, when the process of destruction is slow, and the deviation from the natural form gradual. In this boy the spinal cord in one place was bent almost at a right angle; it was separated from the abscess only by its own fibrous sheath; the greater part of the lumbar plexus was without any bony protection; and yet there was no interruption of any of the functions of the cord, and there had been from the first hardly any pain, either *at* the seat of mischief, or in other parts reflected *from* it.

The case just narrated was tediously chronic in its course, and seemed to require an extraordinary combination of disease to bring it to a fatal end.

IV.—*A Case of Double Transverse Hemiplegia.* By William Baker, A.M., M.D. (London Lancet, Aug. 25, p. 183.)

The earliest symptom observed in this case, a child, four years of age, was pain in the *back part of the head and neck*, which in a day or two became increased, if the head was held in an erect position. Less than a week after the first manifestation of pain, it was perceived that the *left arm* was paralyzed, and the paralysis gradually extended to the *right leg*, the right arm and left leg being still perfect. Two days afterwards, the *right arm* was invaded, and the paralysis gradually increased and extended to the *left leg*. The paralysis was the most complete in the first invaded limbs; the only power over them was slight movement of the fingers and toes. The right arm and left leg he could flex in a trifling degree, and continued to do so to the last. He died about a month after the appearance of the first symptoms. The intellect throughout was undisturbed; sensation and the sphincters perfect; respirations rather quick, but never embarrassed. The treatment adopted was gently and persistently antiphlogistic; the position recumbent.

Unfortunately, the attending physician made no post-mortem examination, and therefore the most important part of its pathology remains in obscurity. The mischief, Dr. Baker thinks, was not in the brain, for intelligence and facial expression were unchanged, and all the nerves of special sense and also those of common sensation were perfect. When the brain sustains an injury above the decussation in the medulla oblongata, and paralysis is the result, the cause and effect are presumed to be invariably opposite or crossed; but in lesions confined to the spinal cord, the effect is said to be as regularly direct, and probably never crossed. Here we have a paralysis of the four limbs, a well-marked *double transverse hemiplegia*, and in spite of a crossed manner of invasion, Dr. Baker is disposed to place the cause of the malady in the spine. From the history of the approach of the paralysis, with the attendant symptoms, he deems it an idiopathic affection of the spine, and pronounces it spinal apoplexy. The spot in which the heat and tenderness were greatest was diagnosticated as the site of effusion or extravasation. Any effused fluid beneath the pia mater of the medulla might travel from the neck to the cauda equina, and the result would be either a complete extinction of sense and motion, or any other degree of modified lesion of either.

The case of Baron Cuvier is described in connection with that above detailed, as that distinguished man died of a transverse hemiplegia. The post-mortem examinations then made revealed no trace of disease when the spinal column was opened. The brain, pharynx, and œsophagus equally failed to present any morbid condition; in fact, in no part of the body could any change be discovered to which the sufferings and death of this illustrious man could be attributed. His illness was probably the consequence of exhaustion alone. But in the case of the child which we have described above, death was doubtless caused by an extension of inflammation to the brain.

V.—*Apoplectic Affections of the Retina.* Under the care of DR. DIXON, Royal London Ophthalmic Hospital. (London Medical Times and Gazette, June 23, 1860, p. 623.)

The value of the ophthalmoscope is well illustrated in several cases where diagnosis would have been difficult in the absence of this means of assistance. The instrument in fact completed the history of the disease, so far as the eye was concerned, and furnished information which formerly could only have been obtained by actual dissection. In *Case* 1, the patient noticed while at work that there was "a dimness before his sight," and, on closing the eyes alternately, he found that he could not see with the left. The next morning the right eye failed in the same manner, and, on further trying the eye, he could only make out very large capital letters. Ten days afterwards vision in his right eye had improved, but in the left it was not materially affected. In strong light he could not see at all. The ophthalmoscope showed traces of effusion from the choroid beneath the retina, at the yellow spot in both eyes, the most in the left. This was a well-marked illustration of symmetrical apoplexy beneath the retina occurring in an apparently healthy man.

*Case* 2 commenced "as a little dimness like a mist before the eye," followed by the appearance of black spots floating before it. His general appearance on admission was not the blank look of total blindness, but he could find his way in the hospital, only slowly, and not without directing himself somewhat by feeling. The pupils were unusually large, and did not contract by light. He could see better with the left eye. The eyes were examined at the time by the ophthalmoscope, and clots of blood were distinctly seen in the fundus. No marked improvement followed the treatment. Constant headache presented a complication in this case, the pain being chiefly in the forehead.

*Case* 3 afforded an example of apoplexy of one retina only, but attended with frontal headache and epistaxis. The failure of sight in the right eye appeared to have followed soon after a violent attack of bleeding from the nose. The headaches were wholly relieved by the epistaxis. When last examined, he described his sensations at night as follows: On looking at the ceiling, he sees a large "block," the size of two heads, the rest of the ceiling appearing pretty clear. (The pupils dilated by atropine.) Upon making an examination of the eye with the ophthalmoscope, a large irregularly circumscribed patch was discovered, extending over and around the yellow spot, the ground of which was lighter colored than the surrounding parts, and on which were numerous dots of extravasation, very irregular, and in many places consisting of five or six spots running into each other. Between this patch and the margin of the optic entrance was found a large and apparently thick coagulum of a deep purple tint. Over the whole of the patch are small white spots, apparently about the size of pins' heads. Several distinct apoplexies must, in this instance, have occurred at different periods. The whitish patches were no doubt those of oldest date. The case is of interest on account of the age of the man being exactly that at which a sister of his had died of cerebral apoplexy, and because in his own case severe frontal headache and epistaxis had preceded the effusion into the eye.

VI.—*Hemorrhagic Diathesis which manifests itself sometimes in the course of Phthisis Pulmonalis and in other Acute or Chronic Affections.* By DR. LEUDET, of Rouen. (Gazette Hebdomadaire, May 25, 1860, p. 350.)

Pathologists have pointed out the occasional coincidence of purpura hemorrhagica and general acute tuberculization, and it has even been attempted to demonstrate that the coincidence is not merely accidental, but that purpura is a symptom of a marked alteration in the circulating fluid itself, the effect of tuberculization. However that may be, we have the testimony of M. Leudet that, in two hundred and forty-four cases of pulmonary consumption, nine exhibited hemorrhagic tendencies from other organs than the lungs. Five times the hemorrhage was from the intestines, twice in the abdominal muscles, twice in the skin, three times in the brain, once by the nose, etc. Simultaneous hemorrhage frequently occurrs from several organs. Entorrhagia connected with tuberculosis of the lungs is not due always to tuberculous ulcerations, for in a majority of cases in which they have been observed, no alteration of this kind had taken place in the intestines.

So also may we place in the same category hemorrhages which have occurred in chronic pleurisy, cancer of the anterior mediastinum, lesions of the kidney, disease of the liver, etc. In cirrhosis of the liver, especially, these hemorrhages the more frequently occur.

VII.—*The Conditions attending every attack of Acute Rheumatism.* By ALBERT T. WHEELOCK, M.D. (American Medical Monthly and New York Review, Aug., 1860, p. 117.)

Dr. Wheelock starts out at once with a proposition of the correctness of which he is fully convinced, by his personal experience—"Every access of acute articular rheumatism is immediately preceded by a special condition of the nervous system induced by mental anxiety or the depressing passions." We quote this startling "truth," as the writer terms it, without indorsing his conviction, that it is, "without [?] egotism, the most important pathological discovery in the present century," founded as it is upon but a small number of cases, fifty in all. The cases furnished by him do not all bear conclusive testimony that the mental condition had any agency as the indirectly exciting cause, or that in the state of mental depression acute rheumatism was invariably the result of a "suppression of the sensible or insensible perspiration," an expression, indeed, which is often used now-a-days, by writers and others, without any very definite idea of its significance.

We cite, by way of illustration, *Case* 1. "A clergyman, aged thirty, married, was suffering much mental anxiety and depression, in consequence of opposition to him, and difficulties in his church. At this period he took a severe cold, *in consequence of being unexpectedly out late at night, thinly clad.* The following day there was a severe attack of acute rheumatism."

*Case* 6. "A clergyman . . . lost by sudden death an only child. . . . . During this mental disturbance, *he became wet in a shower of rain.* . . Acute rheumatism followed."

Dr. Wheelock's observations have settled indubitably, in his opinion, that "mental emotion and cold," (especially cold, we venture to suggest,) "produce with philosophical regularity and mathematical certainty the disease acute rheumatism. The connection between these phenomena is thoroughly proved." He concludes these novel and startling observations by remarking: "In my own experience, I have found, when patients are informed that it has been brought on themselves by a mental agitation that might seem to have been avoided, or was inexcusable and needless, the disease has been shortened in its course or immediately stopped; and where there had been successive attacks, the patients have thus been apparently spared these recurrences." He would deserve the title of the "good physician," who could detect and dispel depressed mental conditions, and thus have it in his power to destroy that most painful disease, acute articular rheumatism.

VIII.—*A Statistical Contribution to the Diagnosis of Cancer of the Stomach.* By JAMES C. ORTON, M.D. (American Medical Times, September 29, 1860, p. 220.)

This paper by Dr. Orton is founded upon an analysis of sixty recorded cases of cancer of the stomach, collected principally from periodical medical literature. In continental cities nearly one-fourth of all the fatal cases of cancers have their origin in the stomach; but no accurate statistics on this point have been registered in this country. Cancer of the stomach is more frequent in old, luxurious cities, than where the style of living is comparatively simple. The *age of greatest liability* to attack is between sixty and seventy; next, between forty and fifty. As regards *the sexes*, males are more subject to it than females, as four to one, and married females to the unmarried, as six to one. We may condense the remainder of Dr. Orton's statistical conclusions embraced in this first contribution on the subject, as follows:—

I. CARDIAC EXTREMITY.—1. *Frequency.*—Less frequent than at the pyloric extremity.*

* Of sixty cases, ten were at the cardiac orifice, sixteen in the body, and thirty-four at the pylorus.

2. *Sex.*—Males more liable than females, as nine to one.*
3. *Age.*—Seldom occurs before the age of fifty.
4. *Duration and Prognosis.*—Rarely extends over several years; the great majority terminate fatally within one year; above one-half do not live beyond six months.
5. *Symptoms.*—Vary with seat, extent, and nature of the cancerous growth.

*a. Dysphagià.*—Diagnostic of the seat of stricture, the cardiac orifice. In the scirrhous variety, may be persistent and intermittent. Attended with severe pain at the pit of the stomach, and threatened suffocation. (In seven cases, five had dysphagia as the first well-marked symptom.)

*b. Pain.*—Caused by arrest of bolus at the strictured point; not constant in character or seat, depending probably much upon the degree of constriction, and occasionally upon the nerves implicated. May be confounded with hepatitis, gastritis, colic, angina, etc. Important when located about the ensiform cartilage; fixed, unvarying, and lancinating, or of a dull, aching character during efforts of deglutition.

*c. Vomiting.*—An early symptom in nine cases out of ten; important diagnostic symptom when promptly following ingestion of food. When very intermittent, disease makes very slow progress, or progresses very rapidly, and is of the encephaloid variety; the freedom from vomiting being due to enlargement of the orifice by ulceration. In the later stages, regurgitation of unaltered food, and vomiting of blood. (Of ten cases, five regurgitated near termination of disease, two had vomiting of blood, etc.)

*d. Tumor.*—Diagnostic of encephaloid. If but slightly hard, and at the ensiform cartilage, probably scirrhous.

*e. Constipation.*—In the earliest stages, from the absence of solid food; and *f. Diarrhœa,* from irritation of the cancerous *debris.*

*g. Emaciation.*—Those cases attended with the greatest degree of emaciation are of the encephaloid variety. (Of ten cases, seven were greatly emaciated.)

II. BODY OF THE STOMACH.—1. *Frequency.*—Intermediate between the orifices.
2. *Sex.*—Males to females as four to one.
3. *Age.*—Occurs about the age of sixty.
4. *Situation.*—In the following order: larger curvature with the great cul-de-sac; the body generally; the smaller curvature.
5. *Form.*—Cancer of the body always encephaloid or colloid, except when seated in the smaller curve.
6. *Duration.*—Several years, generally; and sometimes after long-continued dyspepsia, in which latter case they soon terminate fatally.
7. *Symptoms.*—Confounded at first with dyspepsia. [Dr. Orton's first paper here terminates abruptly, and so, therefore, must our abstract. We may remark, however, that some of the conclusions arrived at appear to be founded upon a very insignificant number of statistical cases.]

---

* Only ten cases are given.

